# The Influence of *Acanthamoeba*–*Legionella* Interaction in the Virulence of Two Different *Legionella* Species

**DOI:** 10.3389/fmicb.2018.02962

**Published:** 2018-12-05

**Authors:** Thiago Santos Gomes, Julia Gjiknuri, Angela Magnet, Lucianna Vaccaro, Dolores Ollero, Fernando Izquierdo, Soledad Fenoy, Carolina Hurtado, Carmen del Águila

**Affiliations:** ^1^Facultad de Farmacia, Universidad San Pablo CEU, CEU Universities, Madrid, Spain; ^2^CAPES Foundation, Ministry of Education of Brazil, Brasília, Brazil

**Keywords:** *Legionella*, *Acanthamoeba*, interaction, virulence, Dot/Icm secretion system

## Abstract

The genus *Legionella* comprises more than 60 species, and about half are associated with infection. *Legionella pneumophila* is the most commonly associated with these infections and by far the most studied, but *L.* non-*pneumophila* species, such as *L. feeleii*, *L. anisa*, etc., may also present clinical importance. Free-living amoebae are their preferred environmental host, where these bacteria not only survive but also succeed in multiplying, and this relationship can lead to an increase in bacterial virulence. The goal of this study was to evaluate the alterations of *Legionella* pathogenicity due to its interaction with *Acanthamoeba*. For this, the expression of protein effectors SdhA, LegK2, and SidK were evaluated in *L. pneumophila* and *L. feeleii*, before and after infecting *Acanthamoeba*. Additionally, the host response was evaluated by measuring the production of IL-6, IL-8, and IFN-γ in infected macrophages. Regarding the virulence factors, an increase in SdhA expression was observed after these bacteria infected *Acanthamoeba*, with a higher increase in the macrophage cultures infected with *L. feeleii*. Also, an increase in the expression of LegK2 was observed after infecting *Acanthamoeba*, but it was more intense in the cultures infected with *L. pneumophila*. With regard to SidK, it was increased in *L. feeleii* after infecting *Acanthamoeba*, however the same effect was not observed for *L. pneumophila*. In cytokine production, the effect on IL-6 and IL-8 was similar for both cytokines, increasing their concentration, but higher production was observed in the cultures infected with *L. feeleii*, even though it demonstrated slightly lower production with the inoculum obtained from *Acanthamoeba*. Concerning IFN-γ, induction was observed in both species but higher in the infection by *L. pneumophila*. Nevertheless, it is not known if this induction is enough to promote an efficient immune response against either *L. pneumophila* or *L. feeleii*. Altogether, these alterations seem to increase *L. feeleii* virulence after infecting *Acanthamoeba*. However, this increase does not seem to turn *L. feeleii* as virulent as *L. pneumophila*. More studies are necessary to understand the aspects influenced in these bacteria by their interaction with *Acanthamoeba* and, thus, identify targets to be used in future therapeutic approaches.

## Introduction

*Legionella* spp. are Gram-negative bacteria with strict growth requirements. The genus comprises more than 60 species that have been isolated from aqueous environments. About half of the known species were also isolated at least once from patients, being thus associated with infection (Cunha et al., 2016; Khodr et al., 2016). Among these species, *Legionella pneumophila* is the most commonly associated with these infections and by far the most studied (Misch, 2016).

Free-living amoebae (FLA) are their preferred environmental host. A large variety of bacterial species have demonstrated their ability to survive and/or multiply within different amoebae, and thus may bypass disinfection treatments when harbored within amoebal cysts, leading to bacterial dispersion into the environment. Due to this feature, amoebic strains, mainly *Acanthamoeba*, have been used in laboratories as host cells to isolate amoeba-resisting bacteria (ARB) from complex samples (Laguna et al., 2006; Corsaro et al., 2009, 2010; Garcia et al., 2013). Moreover, pathogenic bacterial strains emerge from the environment after intracellular replication inside phagocytic protozoans, since bacterial replication within alveolar macrophages seems to mimic the relationship of these bacteria with amoebae. Hence, it has been proposed that its ability to employ basic cellular mechanisms in order to survive numerous protozoan hosts enables *Legionella* to infect human alveolar macrophages and cause disease (Laguna et al., 2006; Hervet et al., 2011; Michard et al., 2015; Sherwood and Roy, 2016). Therefore, the interaction between *Legionella* and FLA could play an important role in the ecology and pathogenicity of these bacteria. However, the number of bacteria isolated from water sources of transmission to humans during outbreaks of Legionnaire’s disease is usually low or undetectable, so an enhanced infectivity of *Legionella* as a consequence of survival and replication within amoebae could compensate for the low infectious dose in the water sources (Richards et al., 2013).

After bacterial uptake, the endosomes containing *Legionella* are reported to follow a different route that bypasses the endocytic pathway and associates with endoplasmic reticulum-derived vesicles, forming the *Legionella*-containing vacuole (LCV), a rough endoplasmic reticulum-like compartment permissive for its intracellular replication. The morphology of the LCV in both mammalian cells and amoebae is similar, which supports the idea that there is an evolutionarily conserved pathway for promoting intracellular growth and suggests that amoeba serves as the “training site” for its ability to colonize higher organisms (Laguna et al., 2006; Zhu et al., 2011; Michard et al., 2015). The formation of the LCV is dependent on a type IVB secretion system (T4SS) known as the defective in organelle trafficking/intracellular multiplication (Dot/Icm) secretion system. This Dot/Icm T4SS translocates more than 300 bacterial proteins, called effectors, into the host cell and is absolutely required to reprogram the endosomal-lysosomal degradation pathway of the phagocytic cell and to trigger the biogenesis of the replication vacuole (Hervet et al., 2011; Harding et al., 2013; Michard et al., 2015).

On the other hand, there is an immunological consequence to this ability to subvert host cell trafficking via secreted effectors. Apart from detecting the pathogen-associated molecular patterns (PAMPs), the innate immune system also detects perturbations of cellular physiology (Misch, 2016). The alveolar epithelial cells and macrophages play a critical role in preventing infection in mammalian lungs, sensing pathogen presence and inducing cytokine secretion to cause inflammation. However, proinflammatory cytokine production is a double-edged sword for the host, it works as a defense response when produced moderately but, when produced excessively, it may act as a pathogenic factor. After all, the pathogenesis of pneumonia is due to both host cell injury by bacterial virulence and its ability to induce host inflammation (Wang et al., 2015).

Previous environmental studies conducted on waters from drinking water treatment plants (DWTP) of Central Spain detected a high environmental presence of *Acanthamoeba* spp. in this region. Moreover, the presence of *Legionella* spp. was also reported in some of these samples. Although it did not detect the presence of *L. pneumophila* in these water samples, the presence of *L.* non-*pneumophila* species was reported both in raw and treated water. Most of these detections were succeeded by polymerase chain reaction (PCR), but an isolate of *L. feeleii* was able to be recovered by co-culture with *Acanthamoeba* (Magnet et al., 2012, 2015). Therefore, the successful isolation of viable *L. feeleii* bacteria led to a significant interest in understanding if the environmental presence of this species could represent a risk. After all, *L. feeleii* has already been reported as a causative agent of both Pontiac Fever and Legionnaire’s disease (Muder and Yu, 2002; Siegel et al., 2010). This previous isolation of viable *L. feeleii* in water through amoebae co-culture and its reported capability of causing disease encouraged us to comparatively analyze some aspects of the virulence of *L. pneumophila* and *L. feeleii*, and also the possible alterations due to their interaction with *Acanthamoeba*.

## Materials and Methods

### Bacteria

Two strains of *Legionella* were used in this study: (i) *L. pneumophila* strain JR32 and (ii) *L. feeleii*, environmental isolate from natural pool, obtained in a previous study (Magnet et al., 2015). They were cultured in AYE medium (Feeley et al., 1979), with some modifications. The composition is as follows: 10.0 g of yeast extract, 55 mM ACES, 3.3 mM L-cysteine, 0.6 mM Fe(NO_3_)_3_.9H_2_O and 5 μg/mL Chloramphenicol. Final pH was adjusted to 6.9. Both strains were cultured in orbital agitation for 22 h at 37°C, and dilutions were prepared when the culture reached OD_600_ ≥ 3. These conditions assured bacteria were at stationary growth phase, characterized for its higher infective capacity.

### *Acanthamoeba* spp.

*Acanthamoeba* ATCC 30234, a genotype T4 strain free of *Legionella* was used in this study. This strain was cultured in axenic PYG medium (0.75% proteose peptone, 0.75% yeast extract, and 1.5% glucose with 40 μg gentamicin per milliliter) at 28°C without shaking.

### Cell Culture

RAW 264.7 (ATCC^®^ TIB-71^TM^), a cell line of murine macrophages, was used in this study and cultured in RPMI 1640 medium (Gibco^®^), supplemented with 10% fetal bovine serum (FBS), at 37°C.

### *Acanthamoeba* Infection Assays

The amoebae infection assays were performed as described by Declerck et al. (2007), with some modifications. These assays were carried out in 24-well tissue culture plates where 2 × 10^5^ *Acanthamoeba* trophozoites were placed in 1.0 mL of Neff’s saline per well. In these wells, a negative control without bacteria (Mock) and three different bacterial multiplicities of infection (MOI) were established, for each *Legionella* species: 1, 5, and 10. The plates were incubated at 33°C for 90 min and a subsequent 1-h incubation with gentamicin (100 μg/mL) was conducted to eliminate remaining extracellular bacteria (i.e., bacteria that failed to infect a trophozoite before gentamicin incubation). Then, three washing steps were performed with 1 mL of Neff’s saline per well. This time was set as time zero for post-infection bacterial recovery at 6, 12, 24, and 48 h post-infection (hpi). These assays were in performed in triplicate.

### Preparation of *Legionella* Inocula Recovered From *Acanthamoeba*

After evaluating the most efficient infection conditions observed at the *Acanthamoeba* infection assays, 10^7^ *Acanthamoeba* trophozoites were cultured in a 75 cm^2^ flask with Neff’s saline and an infection procedure with the elected conditions (i.e., hpi and MOI) was conducted with *L. pneumophila* and *L. feeleii*, separately. At the end of the infection step, the flasks were incubated on ice to detach the amoebae from the culture well. Harvested cultures were concentrated by centrifugation (1,000 rpm/10 min) and resuspended in 100 μL, in which a volume of 300 μL of 0.05% Triton X-100 (Sigma-Aldrich Chemie GmbH, Germany) was added to promote amoebic lysis and intracellular bacteria release. From this solution, 100 μL was inoculated in BCYE agar (with L-cysteine) to recover viable bacteria and incubated at 37°C for 3 days or until bacterial colonies were observed.

### Macrophage Infection Assays

These assays were carried out in 24-well tissue culture plates where 2 × 10^5^ cells were placed in 1.0 mL of RPMI 1640 supplemented with 10% FBS, per well. In these wells, a negative control without bacteria (Mock) and four different bacterial inocula were employed to establish infection: *L. pneumophila* from BCYE agar culture plates, *L. pneumophila* inocula recovered from *Acanthamoeba*, *L. feeleii* from BCYE agar culture plates and *L. feeleii* inocula recovered from *Acanthamoeba*. The macrophage infection was performed similarly as described for the *Acanthamoeba* infection assays, but only the most efficient conditions observed in these assays (MOI 1 at the infection step and 24 hpi incubation afterward) were used. Once the infection step was established (bacterial incubation, gentamicin incubation, and washing steps), these plates were incubated at 37°C for 24 hpi. After this incubation, each culture was transferred to a 5.0 mL tube and concentrated by centrifugation at 1500 rpm/10 min, separating supernatant and pellet for further analysis. These assays were in performed in triplicate.

### Determination of Pro-inflammatory Cytokines

For this analysis, the culture supernatant of each well was submitted to an ELISA assay to determine the concentration of pro-inflammatory cytokines IL-6, IL-8, and IFN-γ. These assays were conducted using Mouse IL-6 (Interleukin 6) ELISA Kit – E-EL-M0044 (Elabscience Biotechnology, Co., Ltd.), Mouse IL-8 (Interleukin 8) ELISA Kit and Mouse IFN-γ (Interferon Gamma) ELISA Kit – E-EL-M0048 (Elabscience Biotechnology, Co., Ltd.), according to manufacturer’s instructions. The cytokine amount was determined by comparing absorbance of each well to a standard curve and inferring values using the CurveExpert Basic 1.7 software.

### Determination of *Legionella* Protein Effectors Expression

#### Elected Protein Effectors

To assess the expression of bacterial virulent factors, three protein effectors translocated by the Dot/Icm T4SS were selected due to their reported role in *Legionella*’s infection: SdhA, LegK2, and SidK. The justification for the specific selection of these genes was based on their reported role in this interaction, briefly described as follows. SdhA is one of the few Dot/Icm effectors that are crucial for the replication of *L. pneumophila* in mouse macrophages, as it is required to maintain the integrity of the LCV and thus avoid the activation of the host cell death (Creasey and Isberg, 2012; Harding et al., 2013). LegK2 is a protein factor that plays an essential role in bacterial survival by inhibiting actin polymerization on the LCV, evading endocytic degradation by preventing late endosome/lysosome association with the phagosome (Michard et al., 2015). SidK is a protein effector that inhibits vacuole acidification, interfering with the ability of the host cells to digest pathogens (Xu et al., 2010).

#### RNA Extraction

The culture pellets derived from the macrophage infection assays were resuspended in 1.0 mL of Trizol^®^ Reagent (Ambion, Life Technologies, Carlsbad, CA, United States), and RNA extraction was performed according to manufacturer’s instructions. RNA concentration was measured using a GE NanoVue Spectrophotometer (GE Healthcare Life Sciences, United Kingdom).

#### Reverse Transcription-Polymerase Chain Reaction

A reverse transcription reaction was performed from 120 ng of extracted RNA using the Thermo Scientific RevertAid H Minus First Strand cDNA Synthesis kit (Thermo Fisher Scientific, United States), according to manufacturer’s instructions. Then, the obtained cDNA was used in a conventional PCR reaction designed to amplify specific fragments of the selected protein effector genes. The specific primers designed for these reactions are shown in Table 1. The PCR was performed in a total volume of 50 μL, using 1X Phusion Flash High-Fidelity PCR Master Mix (Thermo Fisher Scientific, United States), specific primers 0.2 μM and 2 μL of cDNA. The PCR reaction was carried out using a GeneAmp^®^ PCR System 9700 thermocycler (Applied Biosystems, United States), and the amplification parameters were as follows: 3 min at 95°C; 40 cycles of 1 min at 95°C, 1 min at 55°C (SidK) or 60°C (SdhA and LegK2), and 2 min at 72°C; with a final extension step of 3 min at 72°C. The amplified product was visualized after electrophoresis in a 2.0% agarose gel. The visualized fragments were quantified by densitometry using Image J software (National Institutes of Health, United States). Additionally, it was performed RT-PCR assays designed to amplify a constitutive gen from mice [glyceraldehyde-3-phosphate dehydrogenase (GADPH)] in order to assure these extracted RNA samples parted from approximately the same number of cells and thus standardize RNA concentration for these comparative analyses between *in vitro* models. Specific primers designed for this reaction were GADPH Forward (5′-TGA GGC CGG TGC TGA GTA TGT CG-3′) and GADPH Reverse (5′-CCA CAG TCT TCT GGG TGG CAG TG-3′).

**Table 1:** Selected protein effectors and the sequence of the specific primers designed.

Protein effector	Primers	Primers sequences	Fragment size
SdhA	SdhA 5′	5′-ATG ATT TCA GAA AAG ATC AAG CT-3′	1,580 bp
	SdhA 3′	5′-TTA TGC GGA TGG CGC TAA TTG GTT T-3′	
			
LegK2	LegK2 5′	5′-ATG GTT TAT TAC ATA AAT TTG AAG-3′	1,420 bp
	LegK2 3′	5′-TTA GCT TGG GCC TCG CAT CAA-3′	
			
SidK	SidK 5′	5′-TTG TCT TTT ATC AAG GTA GGT ATA-3′	580 bp
	SidK 3′	5′-AAA GGC TTA GGC TTT CTT CC-3′	

## Results

### Gene Expression of SdhA, LegK2, and SidK by *Legionella*, Before and After Infecting *Acanthamoeba*

In *Acanthamoeba* infection assays, the expression of these protein effectors was evaluated to determine the most efficient conditions in order to establish the previous *Legionella*–*Acanthamoeba* infection. For both species, the MOI 1 demonstrated to be the best option to recover bacterial inoculum and, at the time of infection of 24 hpi, gene expression demonstrated to occur more intensely (Supplementary Figure S1).

**FIGURE 1:**
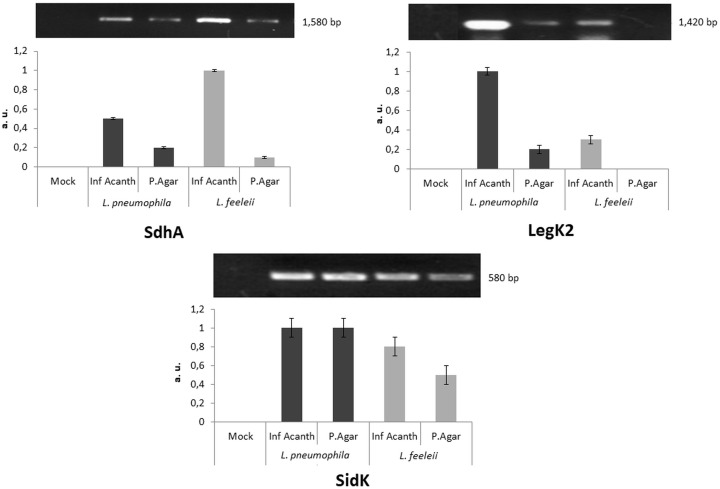
Gene expression of protein effectors SdhA, LegK2, and SidK in macrophage infection, obtained through RT-PCR. The expression of these protein effectors was compared between *L. pneumophila* and *L. feeleii*, with an inoculum from agar culture and another recovered from *Acanthamoeba* infection (bps, base pairs; Inf Acanth, infection of *Acanthamoeba*; Mock, negative control; P. Agar, agar plate; a.u., arbitrary units).

In the macrophage infection assays, RAW 264.7 cells were infected with both species and, for each of the species, an inoculum from agar and another recovered from *Acanthamoeba*. As additional information, a cytopathogenicity analysis and photographs of these macrophage infection assays can be seen in Supplementary Figure S2 and Supplementary Figure S3, respectively. Concerning the gene expression, the results obtained can be seen in Figure 1. In general, these data revealed that, except for SidK, the protein effectors demonstrated to be more intensely expressed at infection by bacterial inocula recovered from a previous infection in *Acanthamoeba* trophozoites. For protein effector SdhA, *L. pneumophila* from agar culture indicated a slightly higher expression than *L. feeleii* from agar. However *L. feeleii* recovered from *Acanthamoeba* demonstrated to not only increase its expression but also in a more intense way than *L. pneumophila* amoebic inoculum. For protein effector LegK2, a higher gene expression was observed in infections by *L. pneumophila* than *L. feeleii*, a result noticed for both agar or *Acanthamoeba* inoculum of each species. For SidK, *L. pneumophila* expression was not altered by the origin of the inoculum while *L. feeleii* expression demonstrated to be higher at the infection produced by the inoculum recovered from *Acanthamoeba*.

### Proinflammatory Cytokines Concentration From Macrophage Cultures Infected With *Legionella*, Before and After Infecting *Acanthamoeba*

**FIGURE 2:**
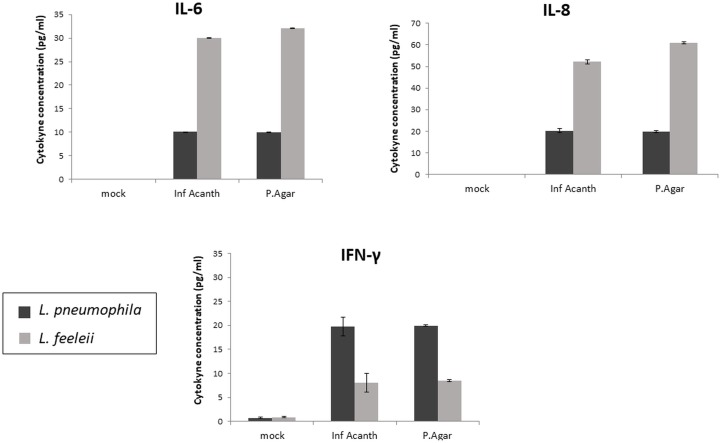
Concentration of cytokines IL-6, IL-8, and IFN-γ in macrophage infection, obtained through ELISA. The production of these cytokines was compared in infections from *L. pneumophila* and *L. feeleii*, with an inoculum from agar culture and another recovered from *Acanthamoeba* infection (Inf Acanth, infection of *Acanthamoeba*; Mock, negative control; P. Agar, agar plate).

The aim of this analysis was to determine possible alterations in inflammatory response from macrophages when infected by *Legionella* from agar culture and by *Legionella* derived from a previous intracellular passage through *Acanthamoeba*. The results can be seen in Figure 2. Regarding IL-6 production, a higher concentration was observed in response to the presence of both species when compared to the uninfected control (Mock), but *L. feeleii* infection stimulated a more intense cytokine production than *L. pneumophila*. Nevertheless, the *L. feeleii* inoculum recovered from *Acanthamoeba* demonstrated to stimulate a slightly lower IL-6 production than bacteria from the agar culture, while *L. pneumophila* inocula recovered from *Acanthamoeba* stimulate similar levels of IL-6 production if compared to infection with bacteria from the agar culture. Regarding IL-8 production, the results were similar to those observed with IL-6. For IFN-γ production, a higher concentration was observed in response to both species presence when compared to the negative control, but *L. pneumophila* infection seemed to stimulate a more intense cytokine production than *L. feeleii*. With regard to the inoculum, its origin did not demonstrate to influence IFN-γ production.

## Discussion

The capability of intracellular bacteria such as *Legionella* spp. to survive and replicate depends on the adequate expression of genes that allow a higher success rate on the establishment of the infection of host cells and for this reason these genes are known as virulence genes. Regarding *Legionella* spp., an important fraction of its pathogenicity is attributed to the activity of effectors translocated by the Dot/Icm T4SS (Zhu and Luo, 2016). Taking into account the virulence produced by these pathogen effectors, three genes belonging to the Dot/Icm T4SS with a described role in *Legionella* intracellular survival were selected for this study.

SdhA is one of the few Dot/Icm effectors that seems to be crucial for the replication of *L. pneumophila* in mouse macrophages, being required for maintaining the integrity of the LCV. Disruption of the vacuole membrane during intracellular replication in macrophages is fatal to both the host cell and the bacterium. Destabilization of the LCV and release of the bacterium into the cytoplasm lead macrophages to rapid cell death due to the activation of pyroptosis, prematurely ending intracellular replication (Creasey and Isberg, 2012; Harding et al., 2013). Unlike apoptosis, which is often anti-inflammatory, pyroptosis is predicted to be proinflammatory, as it leads to a rapid loss of cell membrane integrity and release of cytosolic contents into the extracellular space, exposing pathogens to uptake and efficient killing by neutrophils through the activity of reactive oxygen species (Miao et al., 2010). In this study, SdhA expression was observed as more intense in *L. pneumophila* than *L. feeleii* in the inocula from agar culture, but the passage through *Acanthamoeba* produced a higher increase in the expression of this gene in *L. feeleii* than *L. pneumophila*. These data suggest that infecting *Acanthamoeba* activated the expression of this virulent protein effector in *L. feeleii*, more intensely.

LegK2 is a highly important Dot/Icm T4SS effector that controls actin polymerization remodeling on the LCV, contributing to *Legionella* evasion of endosome trafficking toward the LCV. This evasion of the endocytic pathway is the main virulence-related event of this bacterium infection cycle, preventing fusion with late endosomes and rerouting the phagosome to a replicative niche (Michard et al., 2015). In this study, LegK2 expression was observed as more intense in *L. pneumophila* than *L. feeleii* in both inocula, and the previous passage through *Acanthamoeba* produced an increase in the expression of this gene in these two *Legionella* species. These data could suggest that infecting *Acanthamoeba* increased the expression of this effector, very important in promoting the genesis of the LCV, in both species.

SidK is a protein effector that specifically targets host v-ATPase, the multi-subunit machinery responsible for organelle acidification in eukaryotic cells, and interacts with VatA, a key component of the proton pump. This interaction leads to the inhibition of ATP hydrolysis and proton translocation, decreasing LCV acidification and impairing the ability of host cells to digest its content. SidK expression seems to be highly induced when bacteria begin to enter a new growth cycle (Xu et al., 2010; Michard et al., 2015). In this study, SidK expression was observed as more intensely induced in *L. pneumophila* than *L. feeleii* in the inocula from agar culture. Curiously, the previous passage through *Acanthamoeba* produced an increase in the expression of this gene in *L. feeleii*, but no alterations were observed in the expression of this gene in *L. pneumophila*. Possibly, the ability of *L. pneumophila* to avoid LCV acidification was already an intrinsic characteristic acquired and highly expressed in the *L. pneumophila* strain used in this study, while the environmental *L. feeleii* strain did not express it so intensely and the interaction with *Acanthamoeba* provided an adverse condition in which a higher SidK expression was required for survival.

The higher expression of these protein effectors by inocula recovered from *Acanthamoeba* indicates that this *Acanthamoeba*–*Legionella* interaction might be crucial for increasing the pathogenesis of these bacteria, and these FLA may actually constitute a training site for both *L. pneumophila* and *L.* non-*pneumophila* species. Previous studies compared the invasive ability of bacteria grown in agar culture with that of bacteria grown in *Acanthamoeba* and observed that *L. pneumophila* recovered from *Acanthamoeba* were at least 100-fold more invasive for epithelial cells and 10-fold more invasive for macrophages and amoebae than were *L. pneumophila* grown on agar. Then, it was suggested that these differences could be due to the expression of new proteins in amoeba-grown *L. pneumophila* (Cirillo et al., 1994). This greater invasive ability acquired inside amoebae may be translated in a lower minimal infective dose and, thus, in a higher virulence. Regarding *L. feeleii*, there are no studies comparing virulence traits for this species between isolates from agar culture and from infected amoebae. However, it has already been shown that a *L. feeleii* isolate from a Legionnaire’s disease patient presented a higher invasive capability than another isolate of the same strain of *L. feeleii* recovered from an individual suffering Pontiac fever (Wang et al., 2015). These differences of virulence traits on two isolates of the same strain of *L. feeleii* may be explained by an event that triggered an increase in virulence more intensely in one isolate than the another, and this event could be environmental interaction with free-living protozoa. In these interactions, the protein effectors assessed in the present study may play an important role in promoting a virulence increase, as well as in other effectors that remain to be studied.

Additionally, aspects of the virulence produced by host cells response were evaluated for the alterations produced as a consequence of pathogen activity. First, the proinflammatory cytokines interleukin-6 (IL-6) and interleukin-8 (IL-8) were selected due to their implication in the innate immune response. Cytokine IL-6 is a potent inducer of acute phase protein synthesis, whose levels in bronchoalveolar lavage (BAL) and serum are correlated, suggesting that IL-6 produced in the lung contributes at least in part to serum levels of this cytokine. This is an important feature since systemic levels of IL-6 can be correlated with the severity of the disease (Heinrich et al., 1990; Paats et al., 2013). Interleukin-8 exhibits a potent chemotactic activity for neutrophils and studies also suggest that by chemoattracting neutrophils it indirectly induces CD4^+^ T lymphocyte migration toward the site of infection. Like many inflammatory cytokine responses in community-acquired pneumonia, levels usually are higher in the lungs than in peripheral blood (Mukaida et al., 1998; Paats et al., 2013).

In this study, a similar effect was observed with regard to the production of IL-6 and IL-8 in the infected macrophages. Both cytokine concentrations were increased in the infected cells when compared to uninfected controls and their concentration in the cell cultures was higher in the cultures infected with *L. feeleii*, suggesting that this species might stimulate a more intense response. This could be due to a lower expression of the effectors involved in the escape of the local immune response. Regarding the different bacterial inocula, no alterations in IL-6 or IL-8 concentrations were observed for *L. pneumophila* infection, but a slight decrease in these cytokines concentration were observed in the cultures infected with *L. feeleii* recovered from *Acanthamoeba* when compared with bacterial inoculum from agar. These data suggest that *L. pneumophila* seems to succeed in partially inhibiting the proinflammatory response of the infected cells as described in the literature for the key immune proteins TNF, IL-6, IL-12, and CD86 (Copenhaver et al., 2015), and, for the strain evaluated in this study, this ability does not seem to be increased or decreased after interacting with *Acanthamoeba*. However, *L. feeleii* does not seem to exhibit such an ability in the present study, or at least not to the same degree. Although its passage through amoebae apparently causes a slight decrease on the immune response to its presence, the observed decrease does not reach the low levels produced in response to *L. pneumophila* infection.

The concentration of IFN-γ was also evaluated for its important role in both innate and acquired immunity. An increase of IFN-γ was observed in the presence of both species when compared to the negative control, but a higher increase was observed in the presence of *L. pneumophila*. Regarding the origin of the inocula, no alterations were observed for both *L. pneumophila* and *L. feeleii*. as a consequence of the previous passage through *Acanthamoeba*. Additionally, the observed increase in IFN-γ concentration did not seem to be sufficient to stimulate a proper adaptive immune response, although it is not known if this effect is just a consequence of the lack of participation of other types of cells generating a more systemic type of response. Previous studies demonstrated that human macrophages were able to produce IFN-γ when further stimulated with a combination of IL-12 and IL-18, or with macrophage colony-stimulating factor (M-CSF) (Darwich et al., 2009). In a cell culture containing macrophages exclusively, the co-stimulation generated by cytokines produced by other cells is absent. Previous studies have demonstrated that although macrophages are some of the prototypical producers of IL-12, they do not produce adequate quantities of IL-12 in response to *Mycobacterium tuberculosis*, for example. Furthermore, these studies demonstrated that cytokine interactions were important in restricting the growth of these *Mycobacterium tuberculosis* such us the reciprocal relationship of IFN-γ on IL-18 signaling. IL-18 is known to influence IFN-γ production in a number of cell types in both murine and human systems, but IFN-γ alone also seems to have an effect on IL-18 receptor gene expression, suggesting that other signals received by the macrophage during infection may be necessary to trigger a robust change in the IL-18 receptor at the cell surface (Robinson et al., 2012). Studies have emphasized the importance of the combined synergistic effect of IL-12 and IL-18, both in the differentiation of monocytes into mature macrophages, and in the subsequent stimulation of IFN-γ production, and demonstrated that naturally activated alveolar macrophages immediately secreted IFN-γ upon treatment with IL-12 and IL-18 (Darwich et al., 2009).

## Conclusion

In summary, these data analyzed suggest that the previous passage through *Acanthamoeba*, and possibly other FLA, induce a higher expression of some virulence factors and, thus, the activation of mechanisms to provide the bacterium survival against immune responses. This feature seems to occur in both *L. pneumophila* and *L. feeleii*, but the alterations induced by *Acanthamoeba* appeared more intensely induced in *L. feeleii* than *L. pneumophila*. Thus, these alterations may convert *L. feeleii* to a more virulent bacterium after infecting *Acanthamoeba*. However, this species virulence increase does not seem to make *L. feeleii* as virulent as *L. pneumophila*. On the other hand, the higher immune response stimulated by *L. feeleii* infection may also lead to respiratory clinical symptoms and disease, since proinflammatory cytokine production works as a defense response when produced moderately but also acts as a pathogenic factor if exacerbated. It is important to understand the alterations produced on *L.* non-*pneumophila* species in their environmental interactions to increase awareness of the possibility of infection by those species, generally ignored in laboratorial diagnosis. This study is the first comparative analysis of *L. feeleii* virulence as a result of amoebic interaction and demonstrates the differences observed in this species in comparison to *L. pneumophila*, the most studied species. Nevertheless, more studies are still necessary to understand the entire set of factors influenced in these bacteria as a consequence of their interaction with *Acanthamoeba* and, thus, identify new targets and/or signaling pathways to be used in future therapeutic approaches.

## Author Contributions

TSG, CH, and CdÁ conceived and designed the experiments. TSG, JG, and CH performed the experiments. FI, SF, CH, and CdÁ contributed with reagents, materials, and analysis tools. TSG wrote the first draft of the manuscript. CH and LV wrote sections of the manuscript and prepared figures. All authors analyzed the data, contributed to manuscript revision, and read and approved the submitted version.

## Conflict of Interest Statement

The authors declare that the research was conducted in the absence of any commercial or financial relationships that could be construed as a potential conflict of interest.
