# Supplementary figures and images for: The Influence of Acanthamoeba–Legionella Interaction in the Virulence of Two Different Legionella Species
Source: Front Microbiol. 2018 Dec 5;9:2962. doi: 10.3389/fmicb.2018.02962 (PMC6290054; doi:10.3389/fmicb.2018.02962)

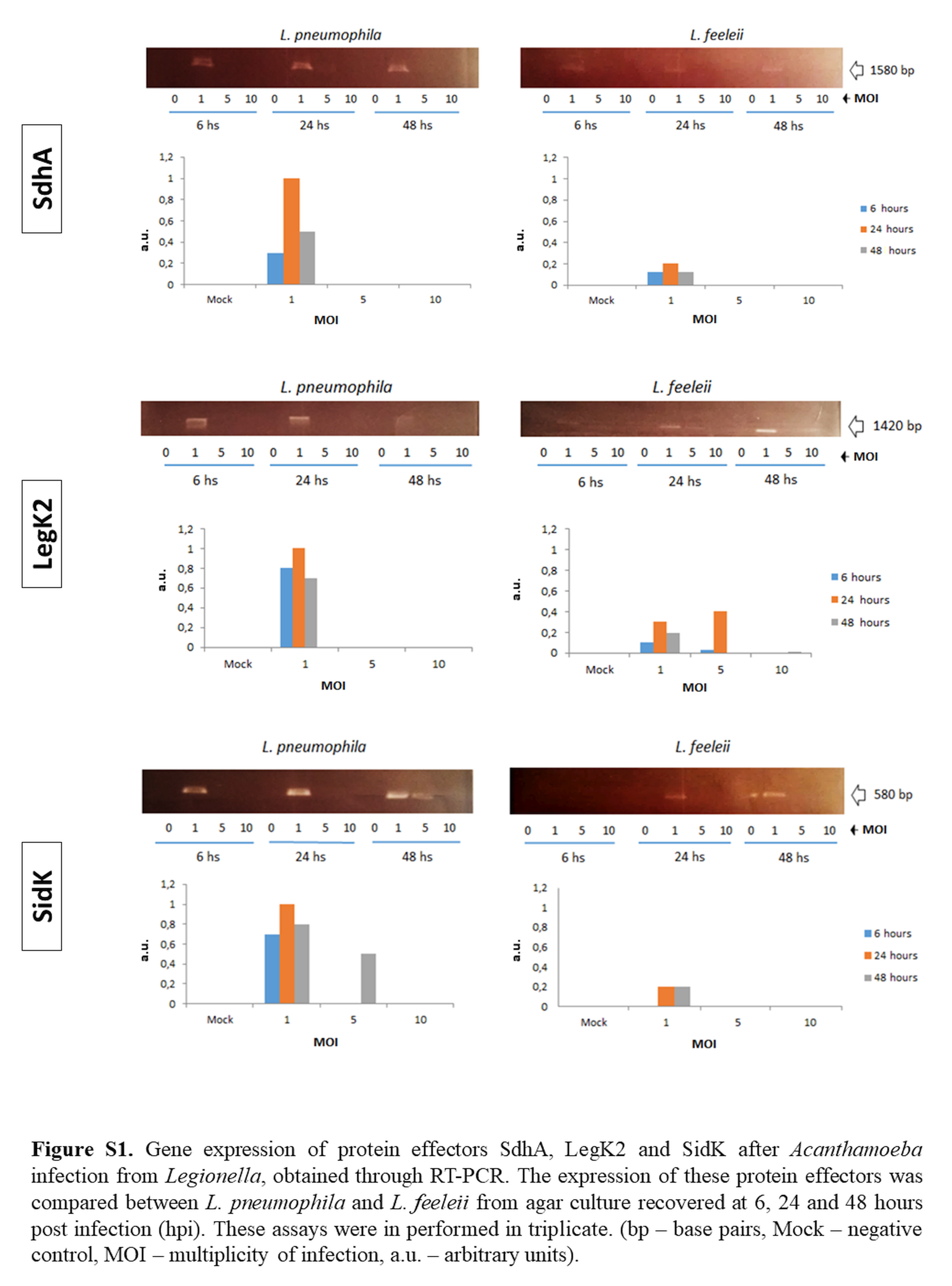

Supplement: Supplementary file 1 [file Image_1.tif]

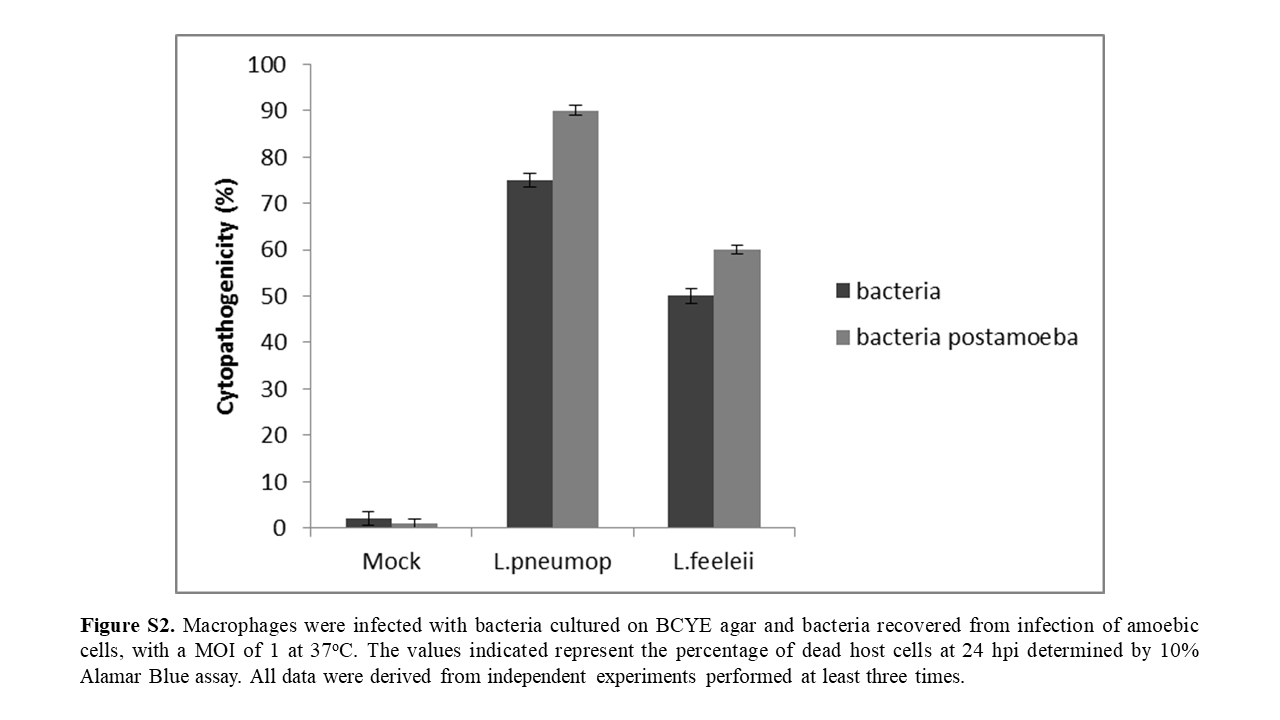

Supplement: Supplementary file 2 [file Image_2.tif]

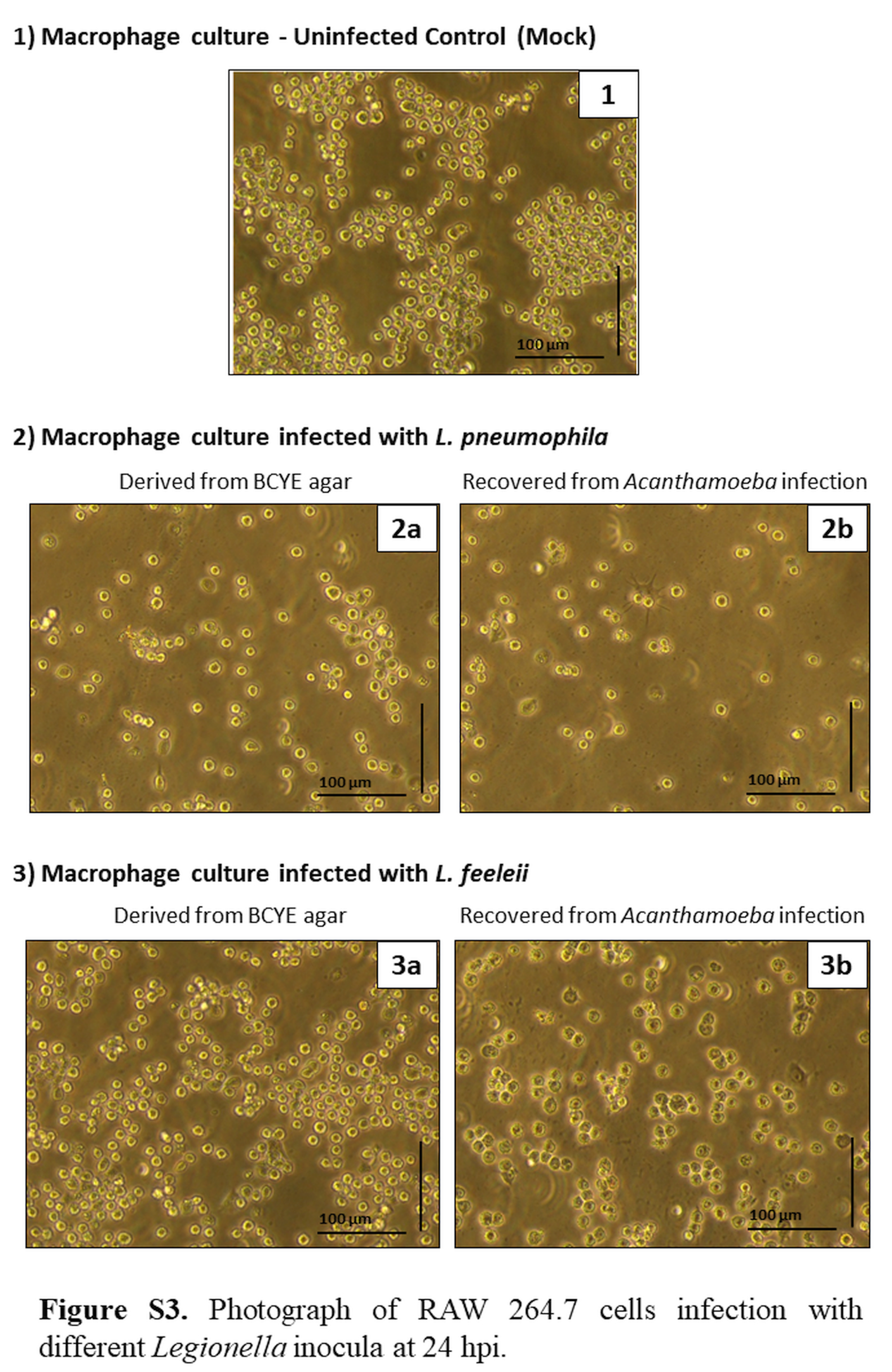

Supplement: Supplementary file 3 [file Image_3.tif]
